# Predation release of Texas horned lizards (*Phrynosoma cornutum*) living in small towns

**DOI:** 10.1002/ece3.7426

**Published:** 2021-03-24

**Authors:** Stephen Mirkin, Mary R. Tucker, Dean A. Williams

**Affiliations:** ^1^ Department of Biology Texas Christian University Fort Worth TX USA

**Keywords:** phrynosoma, pedator‐prey interactions, prey models, urban ecology

## Abstract

Texas horned lizards (*Phrynosoma cornutum*) have a number of ways to avoid predation, including camouflage, sharp cranial horns, flattening of the body, and the ability to squirt blood from the eyes. These characteristics and their relatively low survival rates in the wild suggest these lizards are under high predation pressure. These lizards have been declining in much of their eastern range due to increased urbanization, agriculture, and loss of prey species. However, they can be still be found in some small south Texas towns where they can reach densities that are much higher (~50 lizards/ha) than in natural areas (~4–10 lizards/ha). We hypothesized that one reason for the high densities observed in these towns may be due to reduced predation pressure. We used model Texas horned lizards to test whether predation levels were lower in two south Texas towns than on a nearby ranch. We constructed models from urethane foam, a material that is ideal for preserving marks left behind by predators. Models (*n* = 126) and control pieces of foam (*n* = 21) were left in the field for 9 days in each location in early and late summer and subsequent predation marks were categorized by predator taxa. We observed significantly more predation attempts on the models than on controls and significantly fewer attempts in town (*n* = 1) compared with the ranch (*n* = 60). On the ranch, avian predation attempts appear to be common especially when the models did not match the color of the soil. Our results suggest that human‐modified environments that have suitable habitat and food resources may provide a refuge for some prey species like horned lizards from predators.

## Graphical abstract

Texas horned lizards living in small South Texas towns experience a predation release. This allows them to exist at much higher than normal densities than populations living in more natural environments.

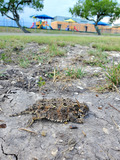

## 1 INTRODUCTION

Urbanization is a major factor in the loss of biodiversity worldwide (Czech & Krausman, 1997; McKinney, 2008; Wilcove et al., 1998). Altered community structure is a hallmark of urban environments; urban communities can differ greatly from their natural counterparts with some species able to adapt, while others decline (Fischer et al., 2012). The role that predation plays in structuring urban communities is not well understood but has been suggested to be similar to what is found in natural areas (Shochat et al., 2006). Increasing evidence, however, indicates that predation may act differently in urban environments, leading to what some authors have termed an urban predation paradox (Eötvös et al., 2018; Fischer et al., 2012; Jokimäki et al., 2020). Studies done largely on urban birds and mammals have shown that urban environments have high densities of mesopredators, but paradoxically lower rates of predation (Eötvös et al., 2018; Fischer et al., 2012). Lower predation rates in urban environments have been attributed to predators subsisting mainly on anthropogenic subsidies (i.e., trash and domestic pet food) (Rodewald et al., 2011). Subsequently, prey species in urban areas may experience an ecological release and lower predation rates that can allow them to exist in hyperabundance (Fischer et al., 2012). Alternatively, some research has shown that predators are more sensitive to urbanization and are pushed out of urban areas because of a lack of suitable habitat. Consequently, urban environments may act as refugia for some prey species due to the lack of predators in those areas (Berger, 2007; Leighton et al., 2010; Muhly et al., 2011; Rebolo‐Ifran et al., 2017; Shannon et al., 2014). With natural habitats being increasingly altered by human development, an important conservation question, now and in the future, will be to determine how predation affects the structure and assemblages of urban communities.

Few studies have been conducted on the impact that predation has on reptiles living in urban environments, and results from these are often conflicting (French et al., 2018). Species richness and abundance are generally negatively correlated with urbanization (McKinney, 2008); however, some studies show that reptiles thrive in urban environments and even increase in abundance and diversity under certain conditions (Barrett & Guyer, 2008; Moreno‐Rueda & Pizaarro, 2007; Schlauch, 1978). Due to the difficulty in observing predation events, many researchers have turned to the use of clay or foam models to measure predation (Bateman et al., 2016). Of these studies, only Mcmillan and Irshick (2010) explicitly tested differences in predation rates between urban and natural environments. Their results, consistent with the urban predation paradox, showed significantly lower amounts of predation on green anole models (*Anolis carolinensis*) in the urban area.

Texas horned lizards (*Phrynosoma cornutum*) are highly specialized lizards with unique morphological characteristics and dietary preferences and exhibit a variety of adaptations for life as myrmecophagus, sit‐and‐wait predators, living in arid environments (Pianka & Parker, 1975; Sherbrooke, 2003). Many of these behavioral and morphological adaptations can be attributed to selective forces in response to predation (Edmunds, 1974; Endler, 1986) and include cryptic coloration, cranial horns, blood squirting, and specific behaviors in response to distinct predators (Middendorf & Sherbrooke, 1992; Pianka & Parker, 1975; Sherbrooke, 1987, 2008). Texas horned lizards are known to have a multitude of predators including snakes, predatory lizards, birds, rodents, canids, and felids (Sherbrooke, 2003). Their low annual survival rate (8.9%–54%) is also often attributed to high predation pressure (Endriss et al., 2007; Fair & Henke, 1999; Miller et al., 2020).

Texas horned lizards, an iconic vertebrate of the American southwest, have declined, especially in eastern areas of their historic range, and are now a threatened species in the state of Texas (Donaldson et al., 1994; Texas Conservation Action Plan—TCAP, 2012). Declines in these once common lizards are attributed to a variety of factors including urbanization and habitat conversion, invasive red fire ants (*Solenopsis invicta*), which can prey on the eggs and young of horned lizards, the loss of harvester ants (*Pogonomyrmex* spp) due to widespread use of insecticides and competition with fire ants, and over‐collecting for the pet and curio trades (Donaldson et al., 1994; Henke, 2003). Texas horned lizards are still found in some small Texas towns, including populations occurring in the towns of Kenedy and Karnes City in southern Texas. Past research has shown that lizards in these towns occur at average densities of 52.32 ± 11.2 SE lizards/ha (Ackel, 2015), which is much higher than the reported densities in more natural areas (3–10 lizards/ha) (Whitford & Bryant, 1979; Whiting et al., 1993). Lizards in these towns predominately eat smaller ants (*Pheidole* spp.) and termites (*Tenuirostritermes cinereus*) rather than their commonly preferred prey of large harvester ants (Alenius, 2018). Foraging on smaller prey items may increase handling time for Texas horned lizards, which would put them at higher risk of predation. We hypothesized that predation rates are lower in town than in more natural areas, and that this has facilitated both high densities of lizards and the exploitation of small prey items in towns.

In this study, we created foam models of Texas horned lizards and placed them in small towns and a natural rural habitat to test the hypothesis that predation rates would be lower in town. We made hatchling, juvenile, and adult models to determine whether predation rates varied by size and to potentially sample smaller predators that might preferentially attack a small lizard over an adult. We also varied the coloration of the models to determine whether less cryptic models were predated at higher rates and if this differed between urban and rural sites.

## 2 METHODS

### 2.1 Field sites

**FIGURE 1:**
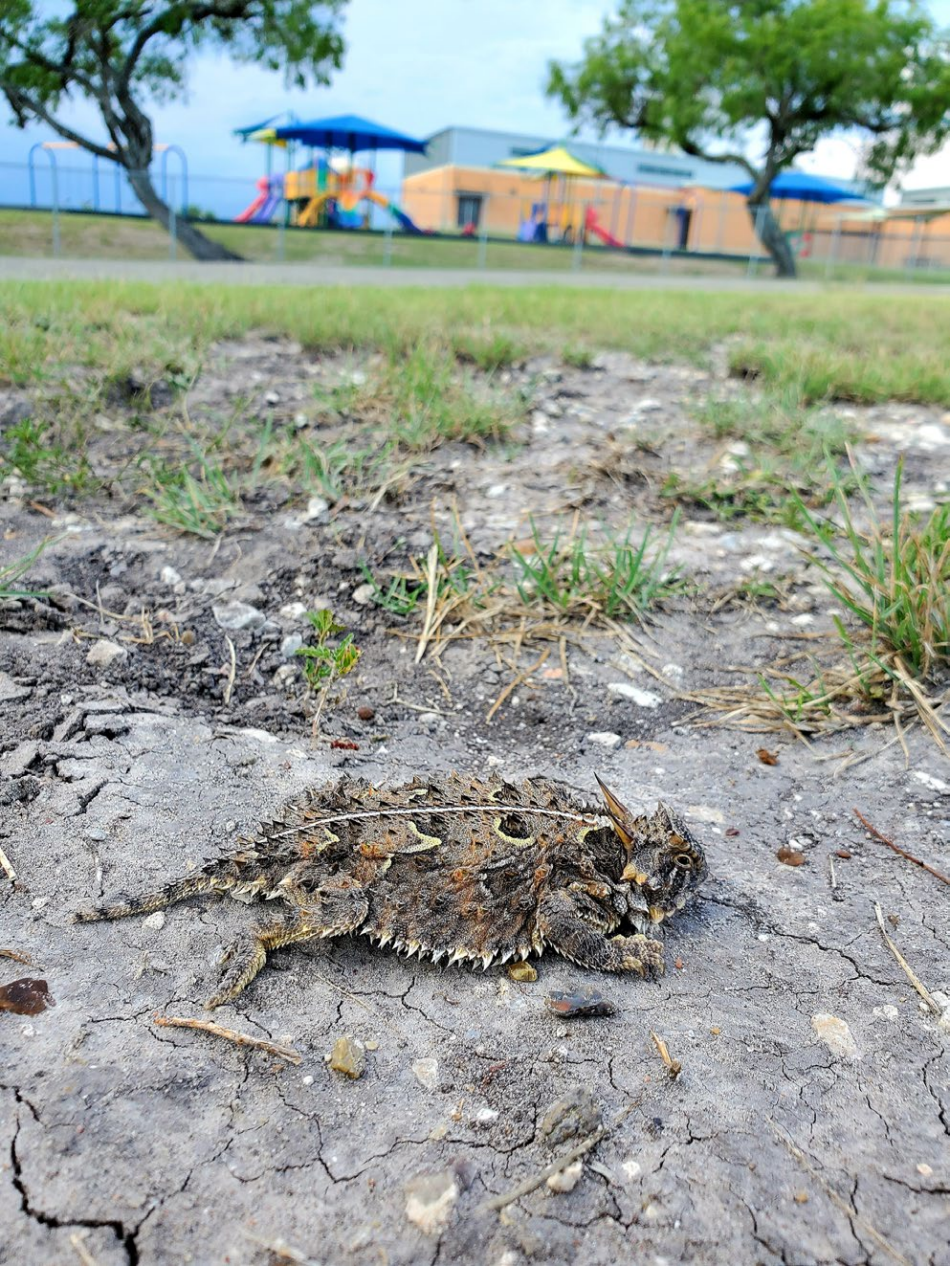
Texas horned lizard near an elementary school in Karnes City, Texas. Photograph by D.A. Williams

We placed models in three locations: Kenedy and Karnes City in Karnes County, Texas, served as urban environments, whereas a private 1,200‐ha hunting ranch in Dimmit County, Texas, served as a natural rural habitat. Karnes City and Kenedy are two small towns (3,299–3,337 people) known for having Texas horned lizards and are the sites of ongoing studies (Figure 1). We have censused 15–17 study plots in Kenedy (3–4 study plots) and Karnes City (12–13 study plots) since 2013 (Ackel, 2015; Alenius, 2018; Wall, 2014). The study plots are irregular in shape, range from 0.054 to 1.22 ha, and represent a variety of suburban habitat types such as alleyways, school yards, vacant lots, parks, and residential areas. Vegetation at all sites consisted of native herbs (especially lamb's‐quarters, *Chenopodium album*; straggler daisy, *Calyptocarpus vialis*; three‐lobed false mallow, *Malvastrum coromandelianum*; and tropical amaranth, *Amaranthus polygonoides*) and grasses (tumble windmill grass, *Chloris verticillata*; plains bristle grass *Setaria vulpiseta*) and the non‐native Bermuda grass (*Cynodon dactylon*). Honey mesquite (*Prosopis glandulosa*), anacua (*Ehretia anacua*), and sugar hackberry (*Celtis laevigata*) are the most common trees on the study plots (Wall, 2014). Each site is surveyed 8–10 times between the end of May and mid‐August. During each survey, we walk linear transects with 2–5 people, spaced 2 meters apart, until we search the entire area of the site. Surveys typically last 20 min to 2.5 hr and are conducted between 0800–1200 and 1600–2000, during active periods for Texas horned lizards.

Over the course of six field seasons (2013–2018), predator observations in and adjacent to our study plots include frequent (daily or weekly) sightings of cats (*Felis catus*), dogs (*Canis lupus familiaris*), and northern raccoons (*Procyon lotor*). We have found very few snakes, including Texas rat snakes (*Elaphe obsoleta lindheimeri*) (*n* = 4 sightings), coachwhips (*Masticophis flagellum*) (*n* = 1 sighting), and bull snakes (*Pituophis catenifer sayi*) (*n* = 1 sighting). Our census methods for horned lizards should be good at detecting snakes on our study plots (e.g., searching through all vegetation, looking under boards, and other fallen objects), and so, even though we did not expect snakes to attack these models (see Discussion) we feel that low snake abundance is a real feature of these towns and so include it here as illustrative of how these towns differ from more natural areas. Predatory birds are also rarely seen, red‐tailed hawks (*Buteo jamaicensis*) (*n* = 3 sightings), greater roadrunners (*Geococcyx californianus*) (*n* = 2 sightings). We have never observed American crows (*Corvus brachyrhynchos*), loggerhead shrikes (*Lanius ludovicianus*), or American kestrels (*Falco sparverius*), in these towns during the summer. American crows are uncommon in this region of Texas and American kestrels are not common during the summer months (Lockwood & Freeman, 2014). Loggerhead shrikes are rare to locally common in Texas (Lockwood & Freeman, 2014) and have been rarely observed on the edge of town and surrounding ranches.

The Dimmit County ranch located within the South Texas Plains ecoregion is approximately 32 km North of the Chapparal WMA (wildlife management area). The habitat is dominated by honey mesquite (*Prosopis glandulosa*) and *Acacia* thornscrub communities typical of south Texas shrubland. This relatively wild habitat maintains natural communities of both predators and prey for Texas horned lizards making it an ideal site for monitoring natural predation on these lizards. Our ad hoc observations of predators on the ranch during the summer of 2018, included a number of potential predators of Texas horned lizards including Harris hawk (*Parabuteo unicinctus*), red‐tailed hawk, Swainson's hawk (*Buteo swainsoni*), American kestrel (*Falco sparverius*), greater roadrunners, and loggerhead shrikes, western diamondback rattlesnake (*Crotalus atrox*), bull snake, Texas indigo snake (*Drymarchon melanurus erebennus*), bobcat (*Lynx rufus*), coyote (*Canis latrans*), and northern grasshopper mice (*Onychomys leucogaster*).

### 2.2 Model construction

**FIGURE 2:**
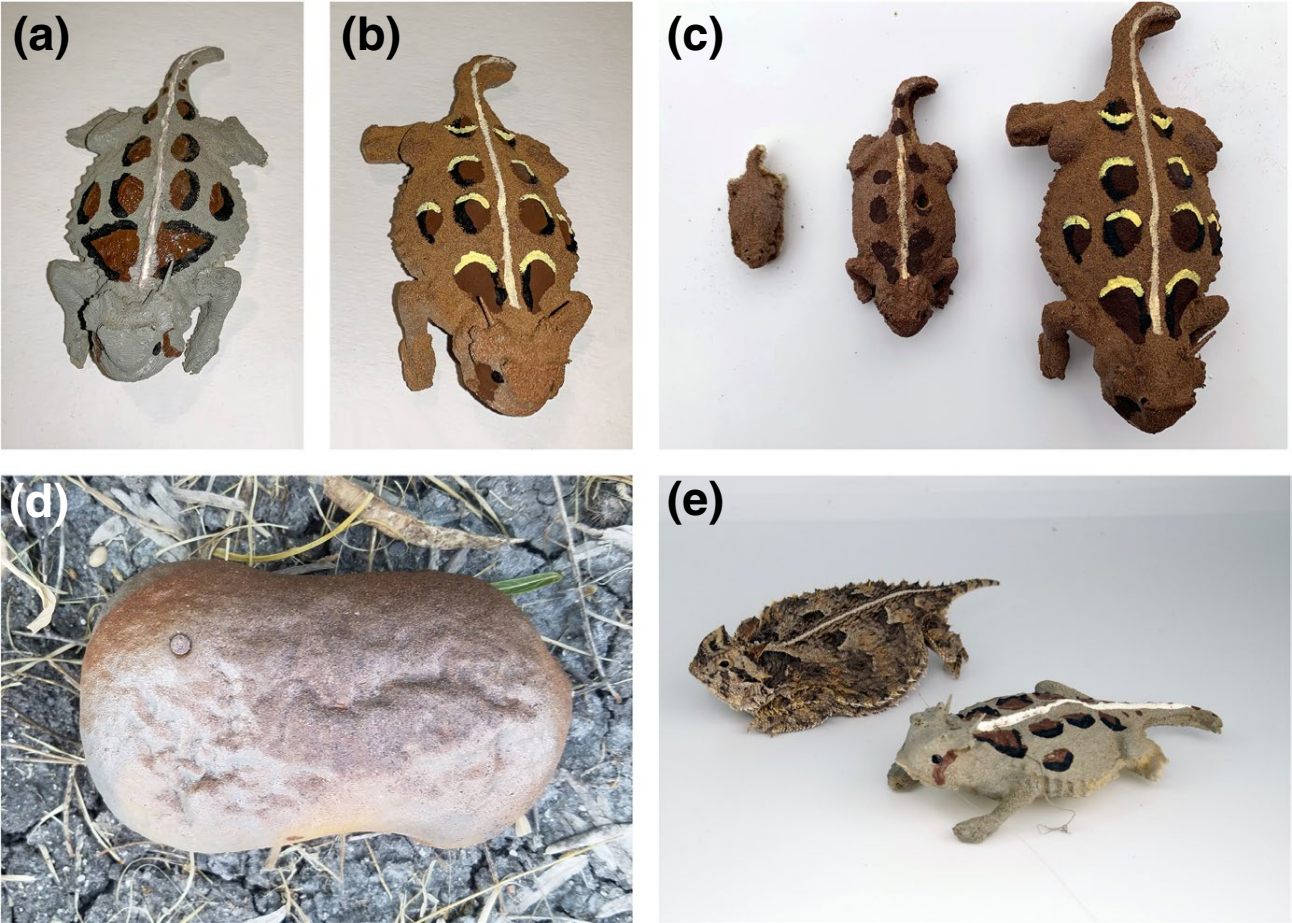
Two distinct color variations of Texas horned lizard models were used in this study. Gray models (a) were painted to match gray lizards found in Kenedy and Karnes City, and red models (b) were painted to match Texas horned lizards found on the Dimmit County ranch. We used 3 size classes of models: hatchlings, juveniles, and adults (c) and controls (d). Models were created to be as morphologically accurate as possible to mimic actual Texas horned lizards (e)

We constructed horned lizard models and controls using urethane foam, a material that has proven effective at withstanding Texas summertime temperatures in excess of 38°C and preserving marks left behind by predation (Farallo & Forstner, 2012). We constructed molds using Mold Max 29NV^®^ silicone rubber (Smooth‐On) and a pewter replica of an adult Texas horned lizard (84 mm snout‐to‐vent length, SVL). The original pewter horned lizard replica was scanned to create an object file (.obj) that was used to 3D print three size classes of model Texas horned lizards: hatchling (23 mm SVL), juvenile (50 mm SVL), and adult (84 mm SVL) size models. We used these 3D printed models to create molds capable of producing the different size classes used in this study and multiple models per casting. Foam iT! 3^®^ urethane foam (Smooth‐On) was poured into the molds and allowed to cure for 2 hr. The controls were constructed from round pieces of urethane foam left over from casting the lizard models and painted with acrylic paint and otherwise treated exactly like the lizard models to control for predators being attracted to the foam material or to the paint (Figure 2).

We painted models and control pieces using acrylic paint to match two distinct color variations of Texas horned lizards (Figure 2); gray lizards from the urban environments of Kenedy and Karnes City where the substrate is varied but predominately gray in color, and red‐colored lizards from a population found in the natural ranch setting in Dimmit County where the substrate is characterized largely by red soils. The specific colors of the models were determined using photographs of multiple adult Texas horned lizards from each location where models were placed, as well as from photographs taken of the surrounding substrates. The PANTONE Studio app (X‐Rite) for iPhone was used to take photographs of the substrates where lizards were previously found to select colors for the models that would accurately resemble local lizards, as well as background color match the substrates. After painting, we placed dried models outside and covered them with a loose layer of soil to allow paint fumes to dissipate for a period of 7 days prior to placing them in the field.

To test whether models were successfully painted to color match their surrounding substrates, we photographed each model in the field with a ColorChecker Passport Photo with software version 1.1.2 (X‐Rite Inc.) in the frame. Using this color standard and the ColorChecker camera calibration software plugin for Adobe Lightroom Classic, we created digital negative (DNG) profiles that could then be used to create images that were calibrated to their true colors, making it possible to compare color values across all photographs. After calibration, a portion of the model lizard's coloration was cropped from the photograph using ImageJ and compared with an exact sized crop of substrate adjacent (~1 cm) to the model. RGB (red, green, blue) color values were obtained from each cropped photograph using the Color Inspector 3D v. 2.3, plugin for ImageJ. These color values were then used to create a color overlapping index (COI) using the COI Function in Rstudio (Samia & Francini, 2015). We calculated COI scores, indicating the percent color match between substrate and model, for 40 models.

### 2.3 Predation experiment

We placed 6 models of each size class (hatchlings, juveniles, adults) and 3 controls across 7 sites in town and 7 sites on the ranch for a total of 126 models and 21 control pieces in both habitat types. The 7 sites in Kenedy and Karnes City included yards, vacant lots, alleyways, and school playgrounds in areas that contained horned lizards determined from previous surveys. The 7 sites of model placement on the ranch included areas with known Texas horned lizard activity as determined by surveys for lizards and their scat. Models were secured to the substrate using 5‐cm nails with the nail head painted over to cover the metallic surface.

We conducted experiments during two‐time intervals: 9 June 2018 to 29 June 2018 and 4 August 2018 to 21 August 2018. During the first 9 days of each period, models were placed in the urban environment and then were subsequently relocated to the natural ranch setting for 9 days. We used these two time periods (early and late summer) to account for differences in weather and possibly predation. During the early summer, models were painted to color match the substrate and lizards in the urban environment. During the late summer, models were repainted to color match the red soils and lizards that were found on the ranch in Dimmit County. This experimental design allowed us to test for any differences in predation rates due to background color matching between models and the —substrate upon which they were placed.

**FIGURE 3:**
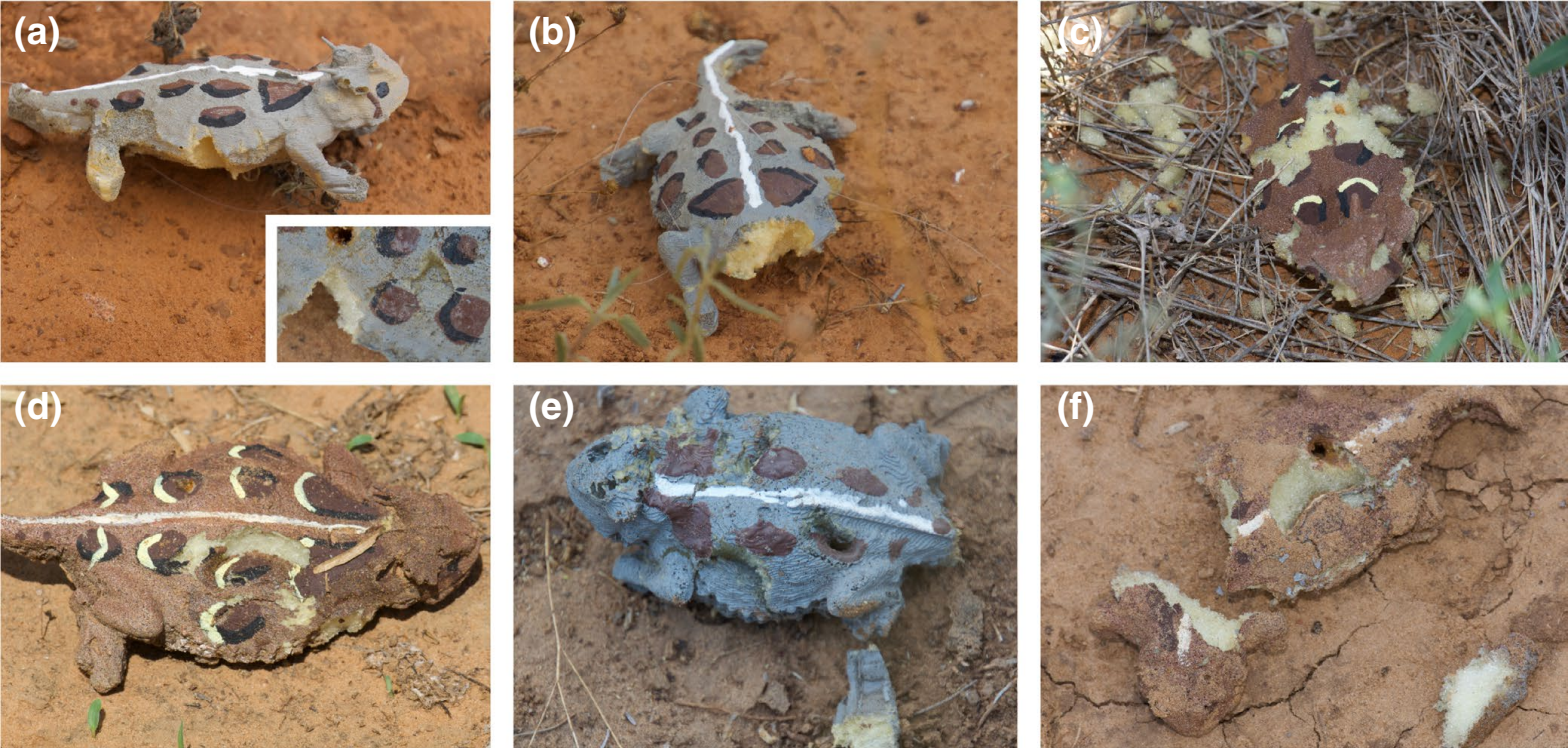
Representative examples of predation events on models attributed to birds (a‐Pecks, b‐Decapitations), Rodents (c), Other (d&e), and Unknown (f)

Upon initial deployment, we recorded the coordinates of each model using the Collector for ArcGIS app (ESRI) with 1‐m accuracy. We also photographed models upon initial placement and upon discovering a potential predation event or disturbance with a Nikon d3300 digital SLR camera with a Tamron 16‐300 mm lens. We checked models every 3 days during the 9‐day period and models that had evidence of predation or disturbance were photographed and removed. We used similar criterion as Brodie (1993) and Bittner (2003) when categorizing predation events. Predation marks on the models were categorized into 4 categories: birds—obvious “V”‐ or “U”‐shaped peck marks and models that had been decapitated; rodents—bite marks with distinguishable chisel teeth imprints left on the model; other—bites that left distinct half‐moon shaped impressions on both upper and lower sides of the model; or unknown—models that had limbs removed but no clear bite or peck marks and models that were found in multiple pieces (Figure 3).

### 2.4 Statistical analysis

For our predation experiment, we compared differences in predation rates of models placed in urban and natural environments using Chi‐square tests when the assumptions of that test were met. Where the assumptions of Chi‐square tests were not met (e.g., expected values of 0) data were then analyzed using Fisher's exact tests. We used two‐sample *t* tests to determine differences in color matching between models and checked for differences between urban gray and urban red models and ranch gray and ranch red models.

## 3 RESULTS

**FIGURE 4:**
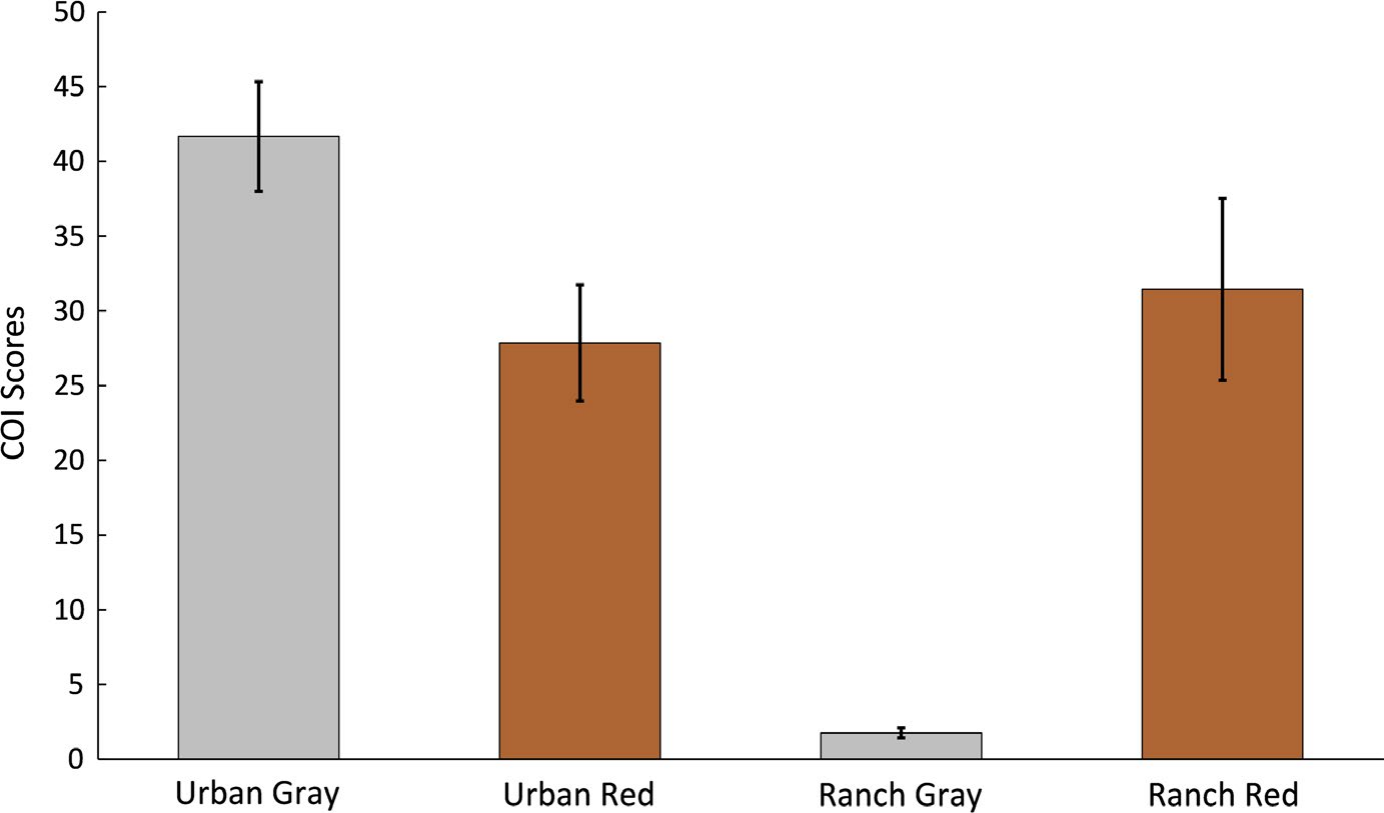
Mean and standard error for COI scores for 10 models in each color‐matching scenario. Models that were painted to background color match their environments have significantly higher COI scores than those that were not painted to match

Models that were painted to color match in the urban environment had higher COI scores than those that were not color‐matched (urban gray versus urban red, t = 2.59, *df* = 18, *p* = .019), and models that were painted to color match on the ranch had higher COI scores than those that were not color‐matched (ranch gray versus ranch red), (t = 4.86, *df* = 18, *p* = .00013) (Figure 4).

A total of 61 predation events were recorded over both sampling periods representing 12.1% of all models. We found no difference in the total number of predation events occurring between early summer (*N* = 30) and late summer (*N* = 31) (*p* = .89) (Table 1). Four predation events occurred on the controls over both sampling periods, all of which were located on the ranch. Predation events were higher on the models than on the controls, both overall (χ^2^ = 3.74, *df* = 1, *p* = .05) and on the ranch (χ^2^ = 3.24, *df* = 1, *p* = .04). We observed more predation events on models at the ranch (*N* = 60) over the two sampling periods than in the towns (*N* = 1) (*p* = 1.12 × 10^−18^) (Table 1). During the early summer, 1 model was attacked in town and 29 were attacked on the ranch (*p* = 1.05 × 10^−8^) (Table 1). During the late summer, no models were attacked in town and 31 were attacked on the ranch (*p* = 1.13 × 10^−10^). At the ranch, hatchling models were attacked less than juvenile or adult models both in the early summer (χ^2^ = 7.08, *df* = 2, *p* = .029) and late summer (χ^2^ = 13.43, *df* = 2, *p* = .001) (Table 1). Models that had evidence of being disturbed by nonpredators (i.e., hoof marks) (*n* = 2) or went missing (*n* = 6) were only on the ranch and were not counted as predation events and excluded from the analysis.

**TABLE 1:** Predation events recorded in town and on the Dimmit County ranch for the two sampling periods and by model size class in 2018

Predation experiment	Urban predation events	Ranch predation events	Total
June (126)	1	29	30
August (126)	0	31	31
Hatchlings (42)	0	6	6
Juveniles (42)	0	26	26
Adults (42)	1	28	29
Controls (42)	0	4	4

Note

Numbers in parentheses are total number of models used at each site.

**FIGURE 5:**
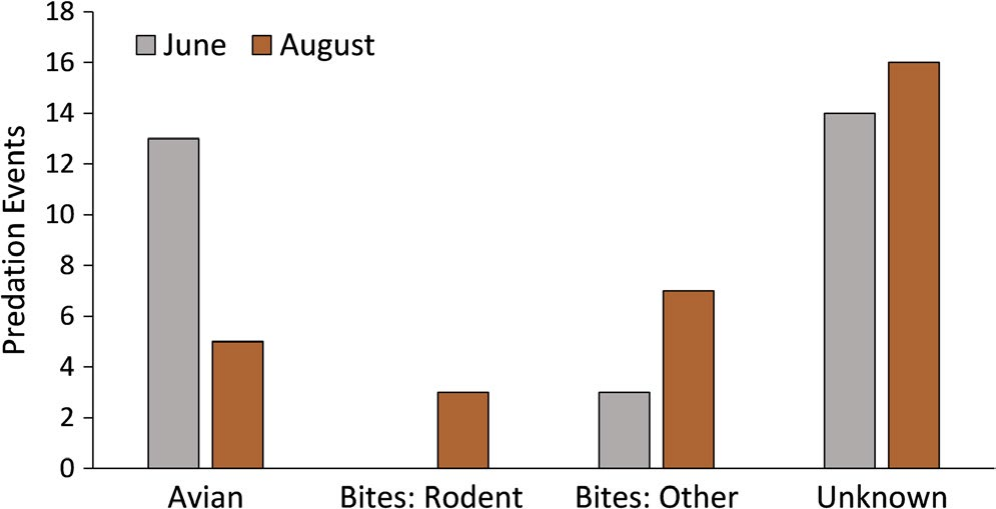
We observed significantly more predation events by avian predators on the ranch when models were not painted to background color match (i.e., were less cryptic) (*p* = .05), suggesting background color matching is an important defense mechanism against predators with high visual acuity. We observed no difference in predation events among the remaining predation categories

We found a significant difference in the number of predation events by predation category at the ranch: birds, rodent, other, and unknown (χ^2^ = 33.24, *df* = 3, *p* = 2.86 × 10^−7^) (Figure 5). We also found that the number of attacks by birds in the early summer (*N* = 13), when lizard models were not painted to color match the red soils on the ranch was significantly higher than during late summer (*N* = 5) when models were painted to background color match (χ^2^ = 3.8, *df* = 1, *p* = .05) (Figure 4). We did not see any difference in color matching in the remaining categories on the ranch rodents: (χ^2^ = 2.9, *df* = 1, *p* = .09), other (χ^2^ = 1.5, *df* = 1, *p* = .23), or unknown (χ^2^ = 0.1, *df* = 1, *p* = .78) (Figure 4). The attack in June on a control piece had distinct peck marks, whereas the three remaining controls that were attacked in August had conspicuous half‐moon shape bite marks. Our controls during the second round were painted to color match the soils on the ranch and as a result resembled dried prickly pear (*Opuntia* spp.) pads and fruits, which may have attracted Texas tortoises (*Gopherus berlandieri*). We frequently encountered Texas tortoises eating both the fruits and pads of *Opuntia* spp. on the Dimmit County ranch and the bite marks on the controls were similar in size and shape to the tortoise bites on cactus pads.

## 4 DISCUSSION

Our results supported our hypothesis that predation on Texas horned lizards in town would be lower than in the natural ranch environment and are consistent with other studies that have found lower predation in urban areas (Fischer et al., 2012). Although we did not explicitly measure predator densities, there is anecdotal evidence that the predator community in Kenedy and Karnes City differs in both the abundance and diversity of predators when compared to the ranch. For instance, birds of prey and snakes are rarely seen in or near our study plots in the towns but were seen daily at the ranch. Feral and pet cats and dogs are also common in town but do not occur on the ranch. Altered predator communities are a consistent result of urbanization (Fischer et al., 2012; Jokimäki et al., 2020; Prange & Gehrt, 2004) and relaxed predation pressure in town may be a contributing factor to the high density of Texas horned lizards in Kenedy and Karnes City (Ackel, 2015) and their dietary shift to consuming small prey items (Alenius, 2018).

Although the use of models has proven effective at measuring predation in other studies (Brodie, 1993; Farallo & Forstner, 2012; McMillan & Irshick, 2010; Vignieri et al., 2010), there are limitations, including the lack of movement, smell, and appropriate behavioral responses, and therefore, models sample a subset of predators and underestimate total predation rates (Bateman et al., 2016). We did not expect models to be attacked by snakes because snakes rely on thermal, motion, and olfactory cues to sense prey (de Cock Buning, 1983); all of which are not exhibited by model lizards. The lack of movement could also decrease model attacks by predators that use motion to detect prey like birds (Antczak et al., 2019) and mammalian predators like cats (Ellis & Wells, 2008). For instance, we set up motion detection video cameras in the urban areas for some models and filmed several instances of cats walking by and ignoring the models. Nevertheless, residents have told us that sometimes their pet cats will bring dead horned lizards back to the house. Cats are known to be very efficient predators of small reptiles, birds, and mammals in urban areas (Loss & Marra, 2017) and stationary model studies may not be well suited to sample these predation events. Another limitation of models may be that predators are attracted to the material used to construct the models (Bateman et al., 2016). Our models were attacked significantly more than the controls however, suggesting predators were not simply attracted to the urethane foam or paint used to construct our models. In the future, we recommend that controls be made into shapes (e.g., pyramids) that would offer no visual cues to encourage predation events such as may have occurred by Texas tortoises mistaking them for cactus pads.

Another common concern in model studies is the confidence with which predation marks left behind on models are accurately categorized. Marks left behind on models by birds were easy to discern by the presence of conspicuous “V”‐ or “U”‐shaped peck marks. Models that had been decapitated were also categorized as avian predation because birds tend to attack toward the head of their prey (Smith, 1973). Avian predators accounted for 30% of predation events. Bite marks accounted for 21.7% and were also relatively easy to discern in models. Bites included obvious chisel marks left behind by rodent incisors and half‐moon bite impressions on the dorsum and venter of models that were possibly indicative of predatory lizards (*Crotophytus reticulatus*.). All other damage to models not falling into those categories were scored as unknown, which represented 48.3% of recorded predation events. These unknown predation events included models that had limbs removed but no definitive bite or peck marks. Northern grasshopper mice (*Onychomys leucogaster*) are known predators of horned lizards (Endriss et al., 2007; Munger, 1986) and have been shown to chew the limbs off Texas horned lizards in areas where both are common (Nathan Rains, Texas Parks, and Wildlife, pers. comm.). We also found several live adult lizards on the ranch with missing limbs, although it is unknown what caused the loss of limbs. The models with missing limbs may also be the result of a typical greater roadrunner “centrifugal‐slam” attack in which the bird grabs a lizard by any limb or tail and smashes it on the ground (Sherbrooke, 1990). These results suggest avian predators are a significant threat to Texas horned lizards, which is similar to findings in other studies that show that avian predators like shrikes consume large numbers of horned lizards and may even be responsible for the evolution of increased cranial horn lengths in flat‐tailed horned lizards (*Phrynosoma mcallii*) (Munger, 1986; Young & Brodie, 2004).

Our hatchling models had fewer predation attempts than the juvenile or adult models. This result may indicate that stationary hatchling lizards are less vulnerable to certain types of predation. Conversely, the hatchling models were morphologically the least realistic, due to their small size and difficulty in constructing them and predators may therefore simply not have recognized them as potential prey.

Birds are highly visual (Fox et al., 1976) and avian predators of horned lizards may use their visual acuity to find their cryptically colored prey. Our results showed that when models were painted to background color match their surroundings (i.e., they were more cryptic), avian predation events were significantly less likely than when models were not painted to color match. This supports a long‐held hypothesis that crypsis and background color matching are the primary defensive adaptations for horned lizards against visually oriented predators (Norris & Lowe, 1964; Pianka & Parker, 1975). A weakness of this result is the confounding of color matching between time periods since all models were painted one color in the early summer and then changed to the other color in the late summer. We did not observe overall predation differences at the ranch between early and late summer nor are there known differences in potential avian predators between these time periods, which could arise for instance, due to migration or breeding. Nevertheless, a better test would be to deploy models with both colors during each period to remove the potential effects of season. Our measure of color matching models to substrate was based on RGB values which only cover a portion of the visual spectrum that potential predators might use. Although digital photographs are good approximations for color variation in this range (Bergeron & Fuller, 2018), there are other important measurements that should be considered when measuring the crypsis of potential prey, such as chromatic and achromatic contrasts modeled after the avian visual system (e.g., Cain et al., 2019). Future studies should explore more fully the color matching of these models and how visible they might be to mammalian and avian predators.

Texas horned lizards have declined throughout their historic range and urbanization is often suggested as one of the main drivers of those declines (Donaldson et al., 1994; Endriss et al., 2007; Wolf et al., 2013). This study presents the first data comparing predation of Texas horned lizards in urban and more natural environments and may serve as a foundation for future studies. Understanding how some horned lizard populations, like those in Kenedy and Karnes City, can persist in urban environments may help inform conservation efforts for other populations. If these human‐modified environments have suitable vegetation and food resources, they may in some cases provide a refuge for some prey species from predators (Keehn & Feldman, 2018; Law et al., 2020). Our results further suggest that Texas horned lizards in natural environments experience high levels of predation pressure which should be an important conservation consideration when targeting areas for potential reintroduction. Models may also be used as a conservation tool in the future by placing them in potential reintroduction areas and in areas where they are established prior to the release of Texas horned lizards to gauge the relative predation pressure the reintroduction site might experience. Knowledge of predation and the predator community at potential reintroduction sites may help increase the probability of survival for reintroduced lizards.

## CONFLICT OF INTEREST

None declared.

## AUTHOR CONTRIBUTIONS

**Stephen Mirkin:** Conceptualization (lead); Data curation (lead); Formal analysis (lead); Funding acquisition (lead); Investigation (lead); Methodology (lead); Project administration (equal); Resources (equal); Software (equal); Supervision (lead); Validation (equal); Visualization (lead); Writing‐original draft (lead); Writing‐review & editing (equal). **Mary Tucker:** Conceptualization (equal); Data curation (equal); Formal analysis (equal); Funding acquisition (equal); Investigation (equal); Methodology (equal); Project administration (equal); Resources (equal); Software (equal); Supervision (equal); Validation (equal); Visualization (equal); Writing‐original draft (equal); Writing‐review & editing (equal). **Dean A. Williams:** Conceptualization (equal); Data curation (equal); Formal analysis (equal); Funding acquisition (equal); Investigation (equal); Methodology (equal); Project administration (equal); Resources (equal); Software (equal); Supervision (equal); Validation (equal); Visualization (equal); Writing‐original draft (equal); Writing‐review & editing (equal).

## ETHICAL APPROVAL

Our Texas horned lizard work was approved by the Institutional Animal Care and Use Committee at Texas Christian University and Scientific Research Permit No. SPR‐0613–073 from Texas Parks and Wildlife.

## Data Availability

The data used in the analysis of this paper can be found in the Dryad Data Repository (https://doi.org/10.5061/dryad.76hdr7svx).

## References

[ece37426-bib-0001] Ackel, A. (2015). The devil in the details: Population estimation for conservation management of Texas horned lizards (*Phrynosoma cornutum*). Masters of Science Thesis, Texas Christian University, Fort Worth.

[ece37426-bib-0002] Alenius, R. (2018). Diet analysis of Texas horned lizards (*Phrynosoma cornutum*). Masters of Science Thesis, Texas Christian University, Fort Worth.

[ece37426-bib-0003] Antczak, M. , Ekner‐Grzyb, A. , Majláth, I. , Majláthová, V. , Bona, M. , Hromada, M. , & Tryjanowski, P. (2019). Do males pay more? A male‐biased predation of common lizard (*Zootoca vivipara*) by great grey shrike (*Lanius exubitor*). Acta Ethologica, 22, 155–162.

[ece37426-bib-0004] Barrett, K. , & Guyer, C. (2008). Differential responses of amphibians and reptiles in riparian and stream habitats to land use disturbances in western Georgia, USA. Biological Conservation, 141, 2290–2300. 10.1016/j.biocon.2008.06.019

[ece37426-bib-0005] Bateman, P. W. , Fleming, P. A. , & Wolfe, A. K. (2016). A different kind of ecological modelling: The use of clay model organisms to explore predator‐prey interactions in vertebrates. Journal of Zoology, 301, 251–262. 10.1111/jzo.12415

[ece37426-bib-0006] Berger, J. (2007). Fear, human shields and the redistribution of prey and predators in protected areas. Biology Letters, 3, 620–623. 10.1098/rsbl.2007.0415 17925272 PMC2391231

[ece37426-bib-0007] Bergeron, Z. T. , & Fuller, R. C. (2018). Using human vision to detect variation in avian coloration: How bad is it? American Naturalist, 191, 269–276. 10.1086/695282 29351010

[ece37426-bib-0008] Bittner, T. D. (2003). Polymorphic clay models of *Thamnophis sirtalis* suggest patterns of avian predation. Ohio Journal of Science, 103, 62–66.

[ece37426-bib-0009] Brodie, E. D. III (1993). Differential avoidance of coral snake banded patterns by free‐ranging avian predators in Costa Rica. Evolution, 47, 227–235. 10.1111/j.1558-5646.1993.tb01212.x 28568087

[ece37426-bib-0010] Cain, K. E. , Hall, M. L. , Medina, I. , Leitao, A. V. , Delhey, K. , Brouwer, L. , Peters, A. , Pruett‐Jones, S. , Webster, M. S. , Langmore, N. E. , & Mulder, R. A. (2019). Conspicuous plumage does not increase predation risk: A continent‐wide test using model songbirds. American Naturalist, 193, 359–372. 10.1086/701632 30794446

[ece37426-bib-0011] Czech, B. , & Krausman, P. R. (1997). Distribution and causation of species endangerment in the United States. Science, 277, 1116–1117.

[ece37426-bib-0012] de Cock Buning, T. (1983). Thermal sensitivity as a specialization for prey capture and feeding in snakes. American Zoologist, 23, 363–375.

[ece37426-bib-0013] Donaldson, W. , Price, A. H. , & Morse, J. (1994). The current status and future prospects of the Texas horned lizard (*Phrynosoma cornutum*) in Texas. Texas Journal of Science, 46, 97–113.

[ece37426-bib-0014] Edmunds, M. (1974). Defense in animals: A survey of anti‐predator defenses (p. 357): Longman.

[ece37426-bib-0015] Ellis, S. L. , & Wells, D. L. (2008). The influence of visual stimulation on the behaviour of cats housed in a rescue shelter. Applied Animal Behaviour Science, 113, 166–174.

[ece37426-bib-0016] Endler, J. A. (1986). Defense against predators. In M. E. Feder , & G. V. Lauder (Eds.), Predator prey relationships: Perspectives and approaches from the study of lower vertebrates: University of Chicago Press.

[ece37426-bib-0017] Endriss, D. A. , Hellgren, E. C. , Fox, S. F. , & Moody, R. W. (2007). Demography of an urban population of the Texas horned lizard (*Phrynosoma cornutum*) in central Oklahoma. Herpetologica, 63, 320–331.

[ece37426-bib-0018] Eötvös, C. B. , Magura, T. , & Lövei, G. L. (2018). A meta‐analysis indicates reduced predation pressure with increasing urbanization. Landscape and Urban Planning, 180, 54–59.

[ece37426-bib-0019] Fair, W. S. , & Henke, S. E. (1999). Movements, home ranges, and survival of Texas horned lizards (*Phrynosoma cornutum*). Journal of Herpetology, 33, 517–525.

[ece37426-bib-0020] Farallo, V. R. , & Forstner, M. R. (2012). Predation and the maintenance of color polymorphism in a habitat specialist squamate. PLoS One, 7, e30316. 22295080 10.1371/journal.pone.0030316 PMC3266262

[ece37426-bib-0021] Fischer, J. D. , Cleeton, S. H. , Lyons, T. P. , & Miller, J. R. (2012). Urbanization and the predation paradox: The role of trophic dynamics in structuring vertebrate communities. BioScience, 62, 809–818. 10.1525/bio.2012.62.9.6

[ece37426-bib-0022] Fox, R. , Lehmkuhle, S. W. , & Westendorf, D. H. (1976). Falcon visual acuity. Science, 192, 263–265. 10.1126/science.1257767 1257767

[ece37426-bib-0023] French, S. S. , Webb, A. C. , Hudson, S. B. , & Virgin, E. E. (2018). Town and country reptiles: A review of reptilian responses to urbanization. Integrative and Comparative Biology, 58, 948–966. 10.1093/icb/icy052 29873730

[ece37426-bib-0024] Henke, S. E. (2003). Baseline survey of Texas horned lizards, *Phrynosoma cornutum* in Texas. The Southwestern Naturalist, 48, 278–282. 10.1894/0038-4909(2003)048<0278:BSOTHL>2.0.CO;2

[ece37426-bib-0025] Jokimäki, J. , Suhonen, J. , Benedetti, Y. , Díaz, M. , Kaisanlahti‐Jokimäki, M.‐L. , Morelli, F. , Pérez‐Contreras, T. , Rubio, E. , Sprau, P. , Tryjanowski, P. , & Ibáñez‐Álamo, J. D. (2020). Land‐sharing vs. land‐sparing urban development modulate predator‐prey interactions in Europe. Ecological Applications, 30(3), e02049. 10.1002/eap.2049 31762100

[ece37426-bib-0026] Keehn, J. E. , & Feldman, C. R. (2018). Predator attack rates and anti‐predator behavior of side‐blotched lizards (*Uta stansburiana*) at southern California wind farms, USA. Herpetological Conservation and Biology, 13, 194–204.

[ece37426-bib-0027] Law, C. , Lancaster, L. , Hale, J. , Handy, S. , Hinchliffee, M. , O'Brien, K. , Watts, S. , & O'Brien, D. (2020). Quantifying the differences in avian attack rates on reptiles between an infrastructure and a control site. European Journal of Wildlife Research, 66, 54.

[ece37426-bib-0028] Leighton, P. A. , Horrocks, J. A. , & Kramer, D. L. (2010). Conservation and the scarecrow effect: Can human activity benefit threatened species by displacing predators? Biological Conservation, 143, 2156–2163. 10.1016/j.biocon.2010.05.028

[ece37426-bib-0029] Lockwood, M. W. , & Freeman, B. (2014). The Texas ornithological society handbook of Texas birds (p. 403): Texas A&M University Press.

[ece37426-bib-0030] Loss, S. R. , & Marra, P. P. (2017). Population impacts of free‐ranging domestic cats on mainland vertebrates. Frontiers in Ecology and the Environment, 15, 502–509. 10.1002/fee.1633

[ece37426-bib-0031] McKinney, M. L. (2008). Effects of urbanization on species richness: A review of plants and animals. Urban Ecosystems, 11, 161–176. 10.1007/s11252-007-0045-4

[ece37426-bib-0032] McMillan, D. M. , & Irshick, D. J. (2010). Experimental test of predation and competition pressures on the green anole (*Anolis carolinensis*) in varying structural habitats. Journal of Herpetology, 44, 272–278. 10.1670/08-196.1

[ece37426-bib-0033] Middendorf, G. A. III , & Sherbrooke, W. C. (1992). Canid elicitation of blood‐squirting in a horned lizard (*Phrynosoma cornutum*). Copeia, 1992, 519–527. 10.2307/1446212

[ece37426-bib-0034] Miller, K. J. , Erxleben, D. R. , Rains, N. D. , Martin, J. C. , Mathewson, H. A. , & Meik, J. M. (2020). Spatial use and survivorship of translocated wild‐caught Texas horned lizards. The Journal of Wildlife Management, 84, 118–126. 10.1002/jwmg.21771

[ece37426-bib-0035] Moreno‐Rueda, G. , & Pizarro, M. (2007). The relative influence of climate, environmental heterogeneity, and human population on the distribution of vertebrate species richness in south‐eastern Spain. Acta Oecologica, 32, 50–58. 10.1016/j.actao.2007.03.006

[ece37426-bib-0036] Muhly, T. B. , Semeniuk, C. , Massolo, A. , Hickman, L. , & Musiani, M. (2011). Human activity helps prey win the predator‐prey space race. PLoS One, 6, e17050.– 10.1371/journal.pone.0017050 21399682 PMC3047538

[ece37426-bib-0037] Munger, J. C. (1986). Rate of death due to predation for two species of horned lizard, *Phrynosoma cornutum* and *P. modestum* . Copeia, 1986(3), 820–824. 10.2307/1444970

[ece37426-bib-0038] Norris, K. S. , & Lowe, C. H. (1964). An analysis of background color‐matching in amphibians and reptiles. Ecology, 45, 565–580. 10.2307/1936109

[ece37426-bib-0039] Pianka, E. R. , & Parker, W. S. (1975). Ecology of horned lizards: A review with special reference to *Phrynosoma platyrhinos* . Copeia, 1975, 141–162. 10.2307/1442418

[ece37426-bib-0040] Prange, S. , & Gehrt, S. D. (2004). Changes in mesopredator‐community structure in response to urbanization. Canadian Journal of Zoology, 82, 1804–1817. 10.1139/z04-179

[ece37426-bib-0041] Rebolo‐Ifrán, N. , Tella, J. L. , & Carrete, M. (2017). Urban conservation hotspots: Predation release allows the grassland‐specialist burrowing owl to perform better in the city. Scientific Reports, 7, 3527. 10.1038/s41598-017-03853-z 28615700 PMC5471179

[ece37426-bib-0042] Rodewald, A. D. , Kearns, L. J. , & Shustack, D. P. (2011). Anthropogenic resource subsidies decouple predator‐prey relationships. Ecological Applications, 21, 936–943. 10.1890/10-0863.1 21639056

[ece37426-bib-0043] Samia, D. S. , & Francini, R. B. (2015). An affordable method to measure animal‐background contrast using digital images. International Journal of Fauna and Biological Studies, 2, 8–16.

[ece37426-bib-0044] Schlauch, F. C. (1978). Urban geographical ecology of the amphibians and reptiles of Long Island. In C. M. Kirkpatrick (Ed.), Wildlife and people (pp. 25–41): Department of Forestry and Natural Resources and the Cooperative Extension Service.

[ece37426-bib-0045] Shannon, G. , Cordes, L. S. , Hardy, A. R. , Angeloni, L. M. , & Crooks, K. R. (2014). Behavioral responses associated with a human‐mediated predator shelter. PLoS One, 9, e94630. 10.1371/journal.pone.0094630 24718624 PMC3981808

[ece37426-bib-0046] Sherbrooke, W. C. (1987). Defensive head posture in horned lizards (*Phrynosoma*: Sauria: Iguanidae). The Southwestern Naturalist, 32, 512–515. 10.2307/3671494

[ece37426-bib-0047] Sherbrooke, W. C. (1990). Predatory behavior of captive greater roadrunners feeding on horned lizards. The Wilson Bulletin, 102, 171–174.

[ece37426-bib-0048] Sherbrooke, W. C. (2003). Introduction to horned lizards of North America: University of California Press.

[ece37426-bib-0049] Sherbrooke, W. C. (2008). Antipredator responses by Texas horned lizards to two snake taxa with different foraging and subjugation strategies. Journal of Herpetology, 42, 145–153. 10.1670/07-072R1.1

[ece37426-bib-0050] Shochat, E. , Warren, P. S. , Faeth, S. H. , McIntyre, N. E. , & Hope, D. (2006). From patterns to emerging processes in mechanistic urban ecology. Trends in Ecology and Evolution, 21, 186–191. 10.1016/j.tree.2005.11.019 16701084

[ece37426-bib-0051] Smith, S. M. (1973). A study of prey‐attack behaviour in young loggerhead shrikes, *Lanius ludovicianus* . Behaviour, 44, 113–141.

[ece37426-bib-0052] Texas Parks and Wildlife Department . (2012). Texas conservation action plan 2012‐2016: Overview. In W. Connaly (Ed.), Texas conservation action plan coordinator: Texas Parks and Wildlife Department.

[ece37426-bib-0053] Vignieri, S. N. , Larson, J. G. , & Hoekstra, H. E. (2010). The selective advantage of crypsis in mice. Evolution, 64, 2153–2158. 10.1111/j.1558-5646.2010.00976.x 20163447

[ece37426-bib-0054] Wall, A. (2014). Home range and genetics of Texas horned lizards (*Phrynosoma cornutum*) in two small towns in south Texas. Masters of Science Thesis, Texas Christian University, Fort Worth.

[ece37426-bib-0055] Whitford, W. G. , & Bryant, M. (1979). Behavior of a predator and its prey: The horned lizard (*Phrynosoma cornutum*) and harvester ants (*Pogonomyrmex* spp.). Ecology, 60, 686–694. 10.2307/1936605

[ece37426-bib-0056] Whiting, M. J. , Dixon, J. R. , & Murray, R. C. (1993). Spatial distribution of a population of Texas horned lizards (*Phrynosoma cornutum*: Phrynosomatidae) relative to habitat and prey. The Southwestern Naturalist, 38, 150–154. 10.2307/3672068

[ece37426-bib-0057] Wilcove, D. S. , Rothstein, D. , Dubow, J. , Phillips, A. , & Losos, E. (1998). Quantifying threats to imperiled species in the United States. BioScience, 48, 607–615. 10.2307/1313420

[ece37426-bib-0058] Wolf, A. J. , Hellgren, E. C. , Bogosian, V. III , & Moody, R. W. (2013). Effects of habitat disturbance on Texas horned lizards: An urban case study. Herpetologica, 69, 265–281. 10.1655/HERPETOLOGICA-D-12-00062.1

[ece37426-bib-0059] Young, K. V. , & Brodie, E. D. (2004). How the horned lizard got its horns. Science, 304, 65. 10.1126/science.1094790 15001716

